# Meta-analysis of the effect and clinical significance of Delphian lymph node metastasis in papillary thyroid cancer

**DOI:** 10.3389/fendo.2023.1295548

**Published:** 2024-01-19

**Authors:** Yan Chen, YiHan Wang, Changlin Li, XueYan Zhang, Yantao Fu

**Affiliations:** Division of thyroid Surgery, China-Japan Union Hospital Of Jilin University, Jilin University, Changchun, China

**Keywords:** Delphian lymph node, thyroid cancer, papillary thyroid carcinoma (PTC), surgical treatment, recurrence

## Abstract

**Objective:**

To investigate the effect and clinical significance of Delphian lymph nodes (DLN) on the factors influencing papillary thyroid cancer (PTC) to provide individualized guidance for the surgical treatment of thyroid cancer.

**Methods:**

Relevant studies from PubMed, Web of Science, the Cochrane Library, Embase, and China National Knowledge Infrastructure databases were searched until February 13, 2023. Stringent selection parameters were used to obtain included data and homogeneous articles. Analyses were performed using Revman 5.4 and SPSS software. A *P*-value of < 0.05 was considered statistically significant.

**Results:**

Five studies were finally included in this study. The results revealed a higher risk of DLN metastasis (DLNM) in patients with tumor size >1cm, multifocality, and extrathyroidal extension (ETE) of the thyroid. The risk of central lymph node metastasis (CLNM) was 11.25 times higher in DLN-positive patients with PTC than in DLN-negative (OR = 11.25, 95% CI: 8.64–14.64, *P* < 0.05) patients. The risk of LLNM was 5.57 times higher in DLN-positive patients with PTC than in DLN-negative (OR = 5.57, 95% CI: 4.57–6.78, *P* < 0.001) patients. The risk of postoperative recurrence in DLN-positive patients with PTC was 3.49 times higher (OR = 3.49, 95% CI: 1.91–6.38, *P* < 0.001) than in DLN-negative patients with PTC.

**Conclusion:**

Patients with tumor size >1 cm in diameter, multifocality, and ETE have an increased risk for DLN development. DLN-positive patients with central and lateral cervical lymph node metastasis and postoperative recurrence are at higher risk than DLN-negative patients.

## 1 Introduction

The global incidence of thyroid cancer has been rapidly increasing in recent times. The global cancer statistics of 2023 (1) reported that thyroid cancer is three times more prevalent in women than in men. Furthermore, papillary thyroid cancer (PTC) is the most prevalent pathological type of thyroid cancer, accounting for approximately 85% ~ 90% (2), which also has a good prognosis and a five-year survival rate of more than 95% (3). Nevertheless, PTC is prone to early regional lymph node metastasis. Previous studies have reported that the rate of lymph node metastasis in patients with PTC ranges from 30%–80% (3–5).

Central lymph nodes (CLN) serve as the primary site for lymph node metastasis of PTC. Within the CLN, the Delphian lymph nodes (DLN) play a crucial role and have the ability to predict the further development of thyroid cancer. The term Delphian was coined by Raymond B. Randall in 1948, a senior at Harvard Medical School. When Raymond B. Randall was an intern at Massachusetts General Hospital, he found that DLN nodules could predict the clinical progression of head and neck malignancies, similar to Delphi the prophet, a priestess of the Temple of Apollo, known for predicting the future (6). It is positioned between the thyroid cartilage and the cricoid cartilage and anterior to the cricothyroid membrane. DLN receives lymphatic drainage mainly from the epiglottis, subglottis, and the superior pole and isthmus of the thyroid gland. Despite its significance, DLN is often easily intraoperatively overlooked by the operator. Therefore, it is not routinely utilized as a central group clearance area.

Ultrasound (US) is the primary method for preoperative diagnosis of lymph node metastasis in thyroid cancer; however, its accuracy in determining central lymph node metastasis (CLNM) is only approximately 10%–63%. US is limited due to its subjectivity and is related to the standard of the operator, contributing to considerable differences in the study results across different institutions (7). Due to the concealed anatomical site of DLN, preoperative US examination is challenging. Hence, pathological evaluations are performed to determine the presence of DLN metastasis (5). Furthermore, lymphatic imaging using the US is a preoperative technique to determine CLN metastases, which can avoid excessive lymph node dissection compared with the intraoperative application of lymph node contrast agent (4); however, the value of its application warrants further investigation.

The postoperative recurrence of patients with PTC has been prevalently reported (8). The presence of lymph node metastasis is strongly associated with the risk of recurrence in patients with PTC (9, 10). Yang et al. (11) reported that the probability of recurrence in patients with PTC was 6.8% and their tumor size was >20 mm. Furthermore, multifocality and cervical lymph node metastasis are independent risk factors for recurrence. Papaleontiou et al. (12) investigated 2454 patients and reported that the probability of postoperative recurrence in patients with PTC was 4.1% and the probability of persistent disease was 5.8%. The differences in the results of the aforementioned studies can be due to the differences in sample size, geography, and other factors. Notably, no large sample study has investigated the relationship between DLN and postoperative recurrence in patients with PTC. To address this gap in literature, we performed a meta-analysis on five studies on influencing factors and clinical significance of DLN metastasis in PTC to investigate the correlation between DLN, lymph node metastasis, and prognosis in patients with PTC.

## 2 Materials and methods

### 2.1 Search strategy

A systematic literature search was conducted to retrieve relevant articles published up to February 13, 2023. We searched PubMed, Web of Science, The Cochrane Library, Embase database, and China National Knowledge Infrastructure (CNKI). The following English keywords were used: (((thyroid carcinoma OR thyroid cancer OR thyroid neoplasm) AND papillary) OR PTC) AND ((prelaryngeal lymph node) OR (Delphian node OR DN) OR (Delphian lymph node OR DLN)). The following Chinese keywords were used in CNKI: (Thyroid Cancer OR Papillary Thyroid Carcinoma) AND (Prelaryngeal Lymph Nodes OR Delphian). The literature selection process was monitored by two data retrievers, with any discrepancies resolved through discussion and consensus or a third author was consulted.

### 2.2 Inclusion and exclusion criteria and literature screening results

The inclusion criteria for this meta-analysis were as follows (1): articles published before March 7, 2023 (2); patients who underwent first thyroid surgery, with pathologically confirmed PTC for tumor and lymph node metastasis; and (3) studies comparing clinical case parameters between positive DLN and negative DLN in patients with PTC.

The exclusion criteria for this meta-analysis were as follows (1): reviews, conference abstracts, commentaries, commentaries, case reports, and (2) studies with insufficient data collection and overlapping clinical data.

**Figure 1:**
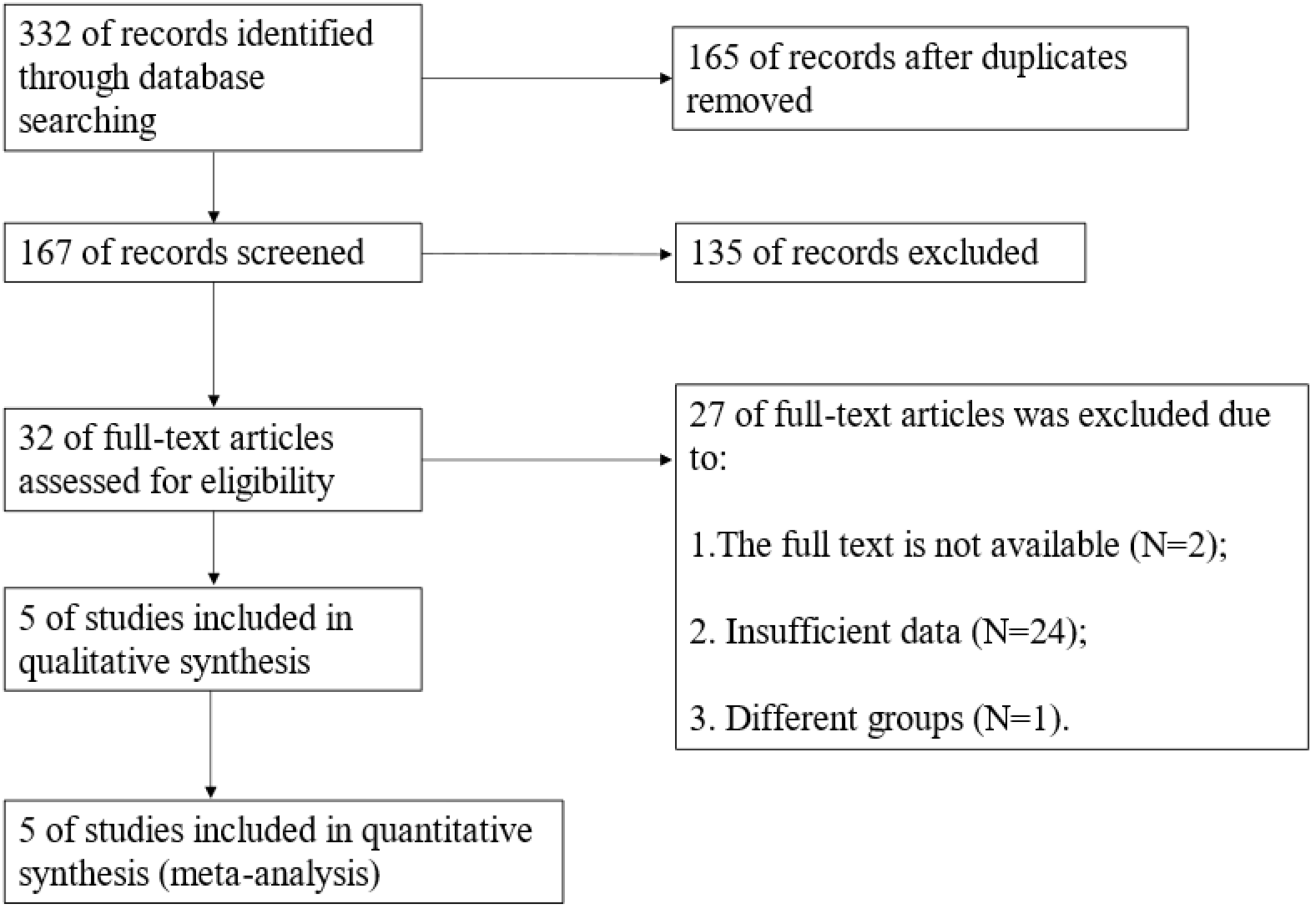
Flow chart of study selection.

The search results yielded a total of 332 articles, including 97 from PubMed, 97 from Web of Science, 3 from The Cochrane Library, 125 from Embase database, and 10 from CNKI. We excluded 165 duplicate articles. Based on primary screening, we excluded 111 articles, and based on conference abstracts, reviews, case reports, and commentaries, we excluded 24 articles. Full text was unavailable for 2 articles. All the articles included in this meta-analysis were not analyzed or grouped differently were 24 articles. One additional study (13) was excluded from this meta-analysis because it included undetected cases of DLN in the DLN-negative group, which is different from other study groups (**Figure 1**).

Finally, based on the Newcastle–Ottawa Scale (NOS) quality evaluation, 5 articles were included (**Figure 1**). Two DLN-related meta-analyses have been previously performed (14, 15). The difference between the two previous meta-analyses and our study is that we have included different indicators. The classification methods of influencing factors of the included articles are different. and the correlation between DLN and recurrence has been investigated. Furthermore, we did not deliberately screen the number and geography of cases.

### 2.3 Data extraction

Two investigators independently evaluated the literature and extracted valid data. Basic information included first author, year of publication, sample size, age (<55 years and ≥55 years), gender, tumor diameter (≤1cm and >1cm), the number of DLN detection and DLN-positive cases, the number of CLNM and LLNM in the subgroup, and the number of recurrences.

### 2.4 Assessment of literature quality

In this meta-analysis, the NOS quality assessment scale was utilized to evaluate the following three aspects: the study population selection, comparability between groups, and outcome indicators. This assessment uses the semi-quantitative principle of the star system, with the exception of comparability, which can be rated up to 2 stars. The rest of the entries can be rated up to 1 star of 9 stars, where higher scores indicate higher study quality. Of note, scores ranging from 0 to 4 were considered as low-quality research, and scores ranging from 5 to 9 were considered as high-quality research (**Table 1**).

**Table 1:** Quality evaluation of the eligible studies with Newcastle–Ottawa scale.

		Chai 2013	Oh 2013	Yan 2021	Zhu 2021	Zuo 2022
Selection	Representativeness	*	*	*	*	*
	Selection of non-exposed	*	*	*	*	*
	Ascertainment of exposure	*	*	*	*	*
	Outcome not present at start	–	–	–	–	–
Comparability	Comparability on most important factors	*	*	*	*	*
	Comparability on other risk factors	*	*	*	*	*
Outcome	Assessment of outcome	*	*	*	*	*
	Long enough follow-up (median≥6 months)	–	–	–	–	–
	Adequacy (completeness) of follow-up	*	–	*	*	–
Quality score		7	6	7	7	6

*indicates criterion met; - indicates significant of criterion not met.

### 2.5 Statistical analysis

The odds ratio (OR) and 95% confidence interval (CI) were calculated using a fixed effects model. Heterogeneity was statistically quantified by performing the I^2^ test. A heterogeneity of I^2^ ≥ 50% was considered significantly different, which was analyzed using a random effects model along with sensitivity analysis. The Revman 5.4 and SPSS software were used to perform statistical analysis. A *P*-value of < 0.05 was considered statistically significant.

## 3 Results

### 3.1 Basic information

**Table 2** presents the basic information. Based on DLN detection, DLN was categorized into negative and positive groups to compare the difference in DLN metastasis rates between the included studies. The results revealed *P* < 0.05, suggesting that the DLN metastasis rates in the five included papers were unequal (**Table 3**).

**Table 2:** Basic information of the 5 included studies.

	Chai 2013	Oh 2013	Yan 2021	Zhu 2021	Zuo 2022
Country	Korea	Korea	China	China	China
Type of study	Retrospective	Retrospective	Retrospective	Retrospective	Retrospective
Institute	Seoul National University Hospital	Gachon University Gil Hospital	Tianjin Medical University Cancer Institute and Hospital	The First Affiliated Hospital of Chongqing Medical University	First People’s Hospital of Yunnan Province
Study period	Jan. 2011 - May 2013	Jul. 2009 - Dec. 2011	Aug. 2017 - Jun. 2020	Jul. 2013 - Dec. 2018	Jan. 2017 - May 2021
Case number (n)	1436	898	516	2271	969
Surgical intervention	TT/LI+CLND±LLND	TT +CLND±LLND	TT/LI+CLND±LLND	TT/LI+CLND±LLND	TT/LI+CLND±LLND
Delphian detection n(%)	370(25.8)	245(27.3)	516 (–)	1575(69.4)	522(53.9)
Delphian Positive n(%)	46(12.4)	49(20.0)	131(25.39)	384(24.4)	106(20.3)

LT, lobe thyroidectomy; TT, total thyroidectomy; CLND, central lymph node dissection; LLND, lateral lymph node dissection.

Table 3Basic information and chi-square tests of DLNM.

**Table:** 

Study * DLN Crosstabulation					
Count					
		DLN		Total	
		Positive	Negative		
Study	Chai 2013 (37)	46	324	370	
	Oh 2013 (16)	106	522	628	
	Yan 2021 (17)	131	385	516	
	Zhu 2021 (14)	384	1191	1575	
	Zuo 2022 (18)	49	196	245	
Total		716	2618	3334

**Table:** 

Chi-Square Tests					
		Value	df	Asymptotic Significance (2-sided)	
Pearson Chi-Square		38.694^a^	4	.000	
Likelihood Ratio		41.136	4	.000	
Linear-by-Linear Association		23.675	1	.000	
N of Valid Cases		3334			

a 0 cells (0.0%) have expected count less than 5. The minimum expected count is 52.62.

### 3.2 Influencing factors

Several original studies have investigated the correlation between age, gender, tumor size, multifocality, extrathyroidal extension (ETE), and DLN metastasis (19, 20). Therefore, this meta-analysis obtained the data to further confirm the correlation between DLN lymph node metastasis and these factors.

#### 3.2.1 Age

**Figure 2:**
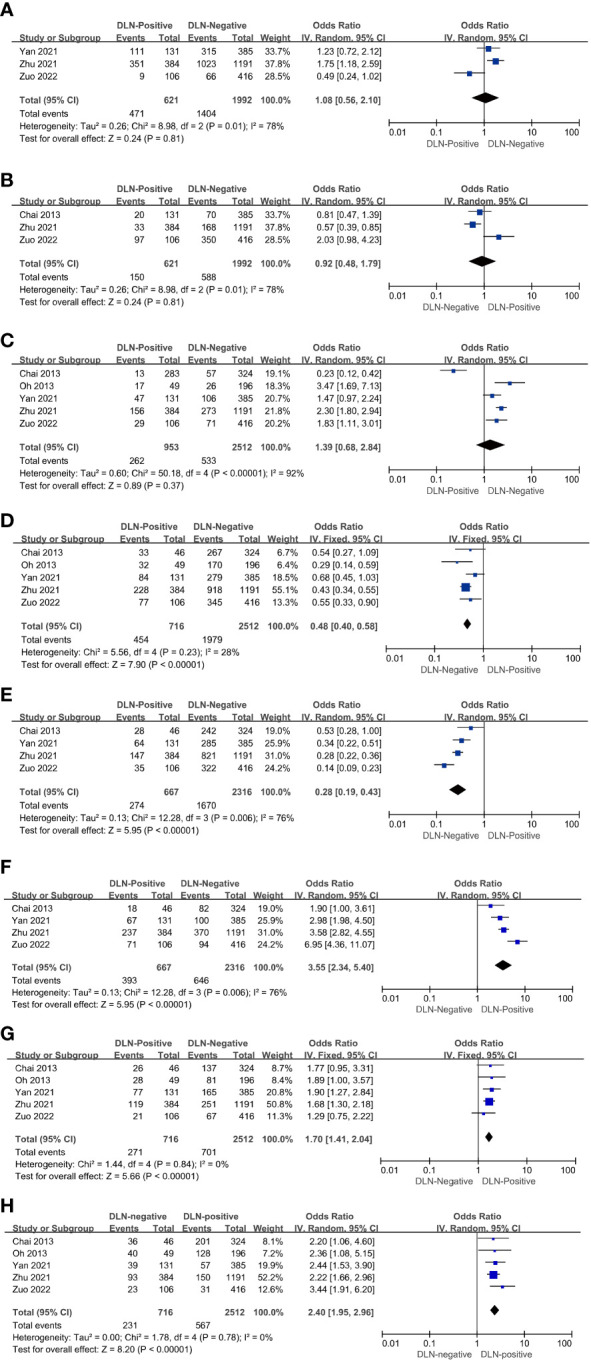
Characteristics with regard to age < 55 **(A)**, age ≥ 55 **(B)**, male **(C)**, female **(D)**, cancer lesion diameter ≤ 1 cm **(E)**, cancer lesion diameter > 1cm **(F)**, multifocality **(G)**, ETE **(H)** of patients with PTC with and without DLNM.

Three of the five included articles reported age-related data. The ages included <55 years (OR = 1.08, 95% CI: 0.56–2.10, *P* = 0.81, **Figure 2A**) and ≥55 years (OR = 0.92, 95% CI: 0.48–1.79, *P* = 0.81, **Figure 2B**) for patients with DLNM transfer, and the results were not statistically significant.

#### 3.2.2 Gender

The results showed that the rate of DLN positivity was 1.39 times higher than DLN negativity in male patients (OR = 1.39, 95% CI: 0.68–2.84, *P* = 0.37, **Figure 2C**). However, with *P* > 0.01, the study did not reach statistical significance. On the other hand, the rate of DLN positivity was 0.48 times higher than DLN negativity in female patients (OR = 0.48, 95% CI: 0.40–0.58, *P* < 0.01, **Figure 2D**). Hence, it can be assumed that DLNM in female patients is less likely to occur.

#### 3.2.3 Tumor size

Four of the five included articles reported tumor size-associated data. The results showed that the DLN-positive rate in patients with tumor size ≤1 cm was 0.28 times higher than DLN-negative (OR = 0.28, 95% CI: 0.19–0.43, *P* < 0.01, **Figure 2E**) patients. Conversely, the DLN-positive rate in patients with tumor size >1 cm was 3.55 times higher than DLN-negative (OR = 3.55, 95% CI: 2.34–5.40, *P* < 0.01, **Figure 2F**). Thus, it can be assumed that DLNM is correlated with the size of the cancer lesion. Furthermore, the risk of DLNM is higher in patients with a cancer lesion diameter >1 cm.

#### 3.2.4 Multifocality

The results showed that the rate of DLN positivity in patients with PTC having multifocal tumors was 1.70 times higher than that of DLN-negative (OR = 1.70, 95% CI: 1.41–2.04, *P* < 0.01, **Figure 2G**). It can be inferred that patients with PTC having multifocal tumors are at a higher risk of DLN positivity.

#### 3.2.5 ETE

The results showed that the rate of DLN positivity in patients with PTC having multifocal tumors was 1.70 times higher than that of DLN-negative (OR=1.70, 95% CI: 1.41-2.04, *P* < 0.01, **Figure 2H**). It can be inferred that patients with PTC having multifocal tumors are at a higher risk of DLN positivity.

### 3.3 Lymph node metastasis

**Figure 3:**
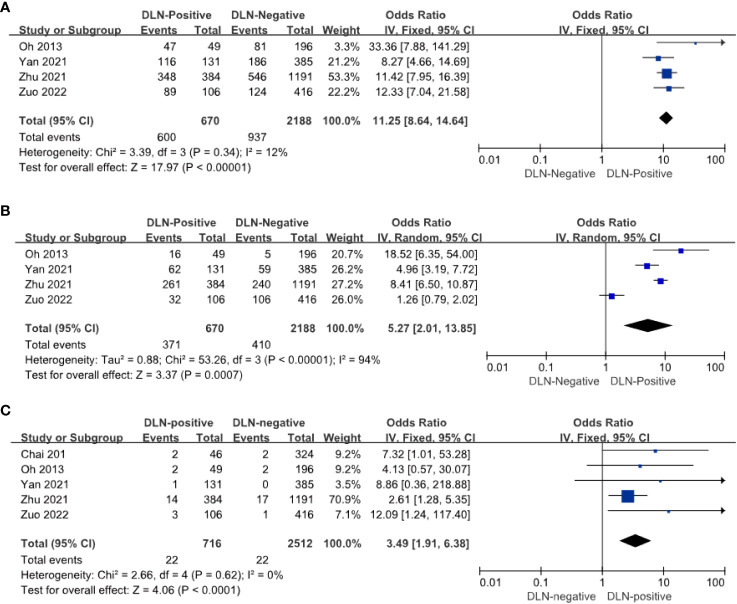
Other compartments regarding CLNM **(A)**, LLNM **(B)**, and recurrence **(C)** of patients with PTC with and without DLNM.

Four of the five articles included relevant data on CLNM and DLN. The results showed that the risk of CLNM in DLN-positive patients with PTC was 11.25 times higher than in DLN-negative patients (OR = 11.25, 95%: 8.64–14.64, *P* < 0.01, **Figure 3A**). The risk of LLNM in DLN positive patients with PTC is 5.57 times higher than DLN negative (OR = 5.57, 95% CI: 4.57–6.78, *P* < 0.01, **Figure 3B**) and it can be assumed that DLNM positive patients are at higher risk of CLNM and LLNM.

### 3.4 Recurrence

Postoperative recurrence occurred in 22 of 716 DLN-positive patients and 22 of 2512 DLN-negative patients. The risk of postoperative recurrence was 3.49 times higher in DLN-positive patients with PTC than in DLN-negative patients (OR = 3.4 9, 95% CI: 1.91–6.83, *P* < 0. 01, **Figure 3C**). It can be reported that DLN-positive patients with PTC have a higher risk of postoperative recurrence.

### 3.5 Heterogeneity analysis

**Figure 4:**
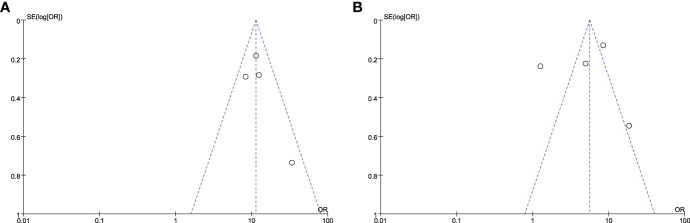
Funnel plot of standard error by log relative risks. **(A)**The CLNM group and **(B)** the LLNM group.

Significant heterogeneity was observed in the forest plots of CLNM (I^2^ = 12%, *P* = 0.34, **Figure 4A**) and LLNM (I^2^ = 94%, *P* < 0.01, **Figure 4B**). Both statistics were analyzed by excluding one study at a time for sensitivity analysis to observe changes in heterogeneity, with no significant changes in heterogeneity in the final results and stable results. Four original studies on CLNM were included, which performed prophylactic clearance of central group lymph nodes. Furthermore, the heterogeneity in the final results may be due to differences in sample size. Heterogeneity in the results of the meta-analysis of LLNM may be due to the different conditions of performing lateral cervical zone clearance. In some studies (13, 16–18), ipsilateral lateral cervical zone lymph node dissection was performed only when preoperative evaluation (US, CT, MRI, or FNA) or intraoperative LLNM was suspected. Additionally, the accuracy of preoperative diagnosis is unknown due to the presence of occult lymph node metastases in patients with PTC. Zhu et al. (14) performed lymph node dissection in the lateral cervical region when all of the following four conditions were simultaneously fulfilled in addition to preoperative diagnosis: intraoperative frozen section finding CLNM ≥ 3, positive DLN metastasis, ETE, and cancer foci located in the upper pole of the thyroid. This led to the heterogeneity in the results of the analysis of the included studies.

## 4 Discussion

No large-sample study has reported the relationship between DLN and postoperative recurrence in patients with PTC. In the present meta-analysis, we examined factors influencing DLN in patients with PTC and performed prognostic analysis by obtaining data from 5 related papers. The results revealed that the risk of DLN metastasis was higher in patients with a cancer diameter >1 cm, multifocality, and ETE. Furthermore, the rate of the central group and lateral cervical lymph node metastasis, along with the risk of postoperative recurrence, were increased in DLNM-positive patients with PTC compared with that in DLNM-negative patients. Therefore, based on this meta-analysis, we believe that when relevant risk factors are identified during the preoperative evaluation of patients with PTC, DLN can be dissected and used for intraoperative rapid pathology analysis. Due to their superficial anatomical position, this neither prolongs the operation time nor increases the risk of nerve or parathyroid injury. When DLN metastases are diagnosed, indicating disease progression, the surgeon should consider the possibility of malignancy in the contralateral lobe or metastasis in the lateral cervical lymph nodes. DLNM-positive patients have a higher rate of postoperative recurrence, suggesting that for patients with intraoperative pathology suggestive of positive DLN metastases, surgery should be more thorough, and these patients should be closely monitored during follow-up. We believe that DLN can have guidance significance for the personalized surgical treatment of patients with PTC.

### 4.1 The special role of DLN

Unlike other CLN, DLNM cannot be diagnosed preoperatively because of some anatomical factors and can only be diagnosed intraoperatively using rapid pathological analysis. Zhao et al. (21) reported that the diagnostic rate of CLNM in the US was 48.0%, and the diagnosis of LLNM was 59.2%. Compared with LLN, CLN is not rapidly diagnosed due to various factors such as sternum occlusion and occult lymph node metastasis. Therefore, prophylactic central group lymph node dissection should be considered in patients with PTC, especially for those with cancer lesions of size > 1cm, multifocal, and ETE. Furthermore, the best treatment plan should be selected after weighing the benefits for patients and the risk of surgical complications. Generally, the lymphatic drainage pattern of thyroid cancer is considered to initially metastasize to the central group and then to the lymph nodes in the lateral cervical region. Nevertheless, Kim et al. (22) reported that male patients, patients with DLN-positive, and patients with CLNM-positive unilateral invasive PTC are at a higher risk of bilateral cervical lymph node metastases. Zuo et al. (18) reported that approximately 10% (4/40) of DLN-positive patients had skipping metastases (only DLNM and LLNM positive and CLNM negative). Liu et al. (23) reported that the incidence of LLNM and skipping metastasis was highest in tumors situated on the glandular lobe, giving a fresh understanding of the nature of lymph node metastasis. We suspected that jumpy metastases are associated with DLNM positivity due to DLN drainage of lymph from bilateral upper thyroid poles and isthmus. Furthermore, the role of DLN in the process of lymph node metastasis in patients with PTC and the mechanism underlying nodal jumping metastasis are unclear and warrant further investigation.

### 4.2 Prognosis and DLN

Patients with PTC tend to experience recurrence after surgery. Postoperative relapses include biochemical recurrence and structural recurrence. The main test for biochemical relapse is serum thyroglobulin (sTg). sTg is presently used to detect persistent disease and recurrent disease (24, 25), with postoperative eugenol suppression treatment sTg < 0.2 ng/mL or TSH-stimulated Tg < 1 ng/mL as the best indicator with the lowest risk of postoperative relapse of 1%–4% (26). In postoperative patients, regular review of Tg helps with the timely detection of recurrence trends and avoids excessive adjuvant testing (27). The following indicators are found to be closely associated with postoperative recurrence: TNM stage, BRAF^V600E^ mutation, postoperative Tg levels, and tumors not completely removed (28, 29). Structural recurrence is a primary recurrence detected in imaging examinations. The US is mainly used for imaging examination of thyroid cancer. FNA, CT, MRI, and 18F-FDG PET can be performed for patients who require further confirmation or for patients with suspected distant metastasis (30). The risk of postoperative recurrence is currently judged postoperatively based on indicators, such as TNM staging. Studies are exploring new potential biomarkers that can be used as prognostic markers in the future. These biomarkers can reveal if preoperative examinations can predict the risk of recurrence among patients, which include investigations on mRNA, microRNA, FN1, ITGα3, MET, miR486, miR-1179, and other genes (31, 32). However, this needs further investigation. Yu Heng et al. (33) reported that patients with PTC situated in the upper portion are more likely to develop LLNM and recurrence. As a part of the central group lymph nodes, the value of DLN in predicting contralateral lobe recurrence or lymph node recurrence in patients with PTC has not been investigated individually. Based on the results of this meta-analysis, we conclude that the risk of postoperative recurrence in DLN-positive patients with PTC is 3.49 times higher than that in DLN-negative patients. Therefore, when combined with the evaluation of preoperative risk factors and intraoperative rapid pathology, if DLN metastasis is detected, lymph nodes should be meticulously dissected. Attention should be focused on re-evaluating whether there is metastasis in the lateral cervical lymph nodes, providing a foundation for individualized intraoperative treatment. There is a notable association between mortality and postoperative recurrence in patients with PTC. Additionally, the probability of surgical complications is significantly increased with secondary surgery in patients (34). Thorough removal of lesions and lymph nodes during the initial surgery, coupled with regular follow-up post-surgery, proves beneficial for patients with PTC. The surgeon must enhance surgical skills and collaborate actively with the patient to achieve the best therapeutic outcomes. Unfortunately, there is a paucity of articles exploring the relationship between DLN and biochemical recurrence or structural recurrence. Due to the lack of more specific data, we anticipate and welcome further prospective studies in the future to contribute to a more comprehensive understanding of this relationship.

### 4.3 Influencing factors of DLN

In present studies on laryngeal and hypopharyngeal cancers, DLNM positivity is considered a risk factor for poor prognosis, indicating an unfavorable outcome for patients (35). Nevertheless, in thyroid cancer, the clinical significance of DLN is not known. Previous studies have reported that anterior laryngeal lymph nodes are the sentinel lymph nodes of thyroid cancer and have a predictive effect on cervical lymph node metastasis. Furthermore, DLNM-positive patients have a higher rate of central group lymph nodes and lymph nodes in the lateral cervical region compared with that in DLNM-negative patients (15, 19, 36). Some original studies have reported that the detection rate of DLN is approximately 25.8%–76% (13, 16, 18, 37) and the metastasis rate is approximately 12.4%–25.4% (13, 14, 16–18, 37). Metastasis in the anterior laryngeal lymph nodes correlated with age, gender, multifocality, metastatic site of paratracheal lymph nodes, and location of cancer foci. These can be considered as risk factors. However, the findings of Gong et al. do not support these findings (20). The results of this meta-analysis confirmed that the factors, namely cancer foci >1 cm in diameter, multifocality, and ETE, can be considered risk factors.

To summarize, the presence of positive DLNM indicates a more extensive lymph node metastasis and a higher risk of recurrence in patients. In clinical practice, operators should prioritize DLN and strive for complete clearance when DLNM is present. Clinically, physicians should be attentive to DLN by evaluating preoperative risk factors and relying on intraoperative DLN analysis for rapid pathology. In the presence of positive DLNM, achieving complete lymph node dissection and maintaining close follow-up should be crucial. DLNM has a guiding significance for the personalized surgical treatment of patients with PTC.

The present study also has some limitations that should be addressed. Based on stringent parameters for data screening, only 5 articles were finally included, of which 3 are from China and 2 are from South Korea. Therefore, the results of this meta-analysis are limited due to lesser geographical coverage. However, it is important to note that the number of cases and geographical location were not deliberately screened in the data screening process. Therefore, DLN in this global region may garner more attention and may benefit from routine testing during surgery to establish individualized treatment. Future studies are expected to encompass a broader range of regions to study DLN, allowing in-depth exploration. Furthermore, even regions with a higher DLN rate can be screened in the future.

## Data availability statement

The original contributions presented in the study are included in the article/supplementary material. Further inquiries can be directed to the corresponding author.

## Author contributions

YC: Writing – original draft. YW: Writing – original draft. CL: Writing – review & editing. XZ: Writing – review & editing. YF: Writing – review & editing.
